# Biosynthesis of caffeic acid in *Escherichia coli *using its endogenous hydroxylase complex

**DOI:** 10.1186/1475-2859-11-42

**Published:** 2012-04-04

**Authors:** Yuheng Lin, Yajun Yan

**Affiliations:** 1 Department of Biological and Agricultural Engineering, the University of Georgia, Athens, GA 30602, USA; 2 Biochemical Engineering Program, Faculty of Engineering, the University of Georgia, Athens, GA 30602, USA

## Abstract

**Background:**

Caffeic acid (3,4-dihydroxycinnamic acid) is a natural phenolic compound derived from the plant phenylpropanoid pathway. Caffeic acid and its phenethyl ester (CAPE) have attracted increasing attention for their various pharmaceutical properties and health-promoting effects. Nowadays, large-scale production of drugs or drug precursors via microbial approaches provides a promising alternative to chemical synthesis and extraction from plant sources.

**Results:**

We first identified that an *Escherichia coli *native hydroxylase complex previously characterized as the 4-hydroxyphenylacetate 3-hydroxylase (4HPA3H) was able to convert *p*-coumaric acid to caffeic acid efficiently. This critical enzymatic step catalyzed in plants by a membrane-associated cytochrome P450 enzyme, *p*-coumarate 3-hydroxylase (C3H), is difficult to be functionally expressed in prokaryotic systems. Moreover, the performances of two tyrosine ammonia lyases (TALs) from *Rhodobacter *species were compared after overexpression in *E. coli*. The results indicated that the TAL from *R. capsulatus *(*Rc*) possesses higher activity towards both tyrosine and *L*-dopa. Based on these findings, we further designed a dual pathway leading from tyrosine to caffeic acid consisting of the enzymes 4HPA3H and *Rc*TAL. This heterologous pathway extended *E. coli *native tyrosine biosynthesis machinery and was able to produce caffeic acid (12.1 mg/L) in minimal salt medium. Further improvement in production was accomplished by boosting tyrosine biosynthesis in *E. coli*, which involved the alleviation of tyrosine-induced feedback inhibition and carbon flux redirection. Finally, the titer of caffeic acid reached 50.2 mg/L in shake flasks after 48-hour cultivation.

**Conclusion:**

We have successfully established a novel pathway and constructed an *E. coli *strain for the production of caffeic acid. This work forms a basis for further improvement in production, as well as opens the possibility of microbial synthesis of more complex plant secondary metabolites derived from caffeic acid. In addition, we have identified that TAL is the rate-limiting enzyme in this pathway. Thus, exploration for more active TALs via bio-prospecting and protein engineering approaches is necessary for further improvement of caffeic acid production.

## Background

Caffeic acid (3,4-dihydroxycinnamic acid) is a natural phenolic compound initially found in plants. Previous studies on its biological activities suggested that caffeic acid possesses anti-oxidant [1,2], anti-virus [3], anti-cancer [4] and anti-inflammatory properties [5]. Moreover, its derivative, caffeic acid phenethyl ester (CAPE), has drawn great attention because of its demonstrated therapeutic effects including its potential as an anti-diabetic and liver-protective agent as well as an anti-tumor drug for human breast cancer treatment [6,7].

Caffeic acid is one of the pivotal intermediates of plant phenylpropanoid pathway starting from the deamination of phenylalanine which generates cinnamic acid. Followed by a two-step sequential hydroxylation at the 4- and 3- position of the benzyl ring, cinnamic acid is converted into caffeic acid via *p*-coumaric acid [8,9]. The involved enzymes, cinnamate 4-hydroxylase (C4H) and *p*-coumarate 3-hydroxylase (C3H) are plant-specific cytochrome P450 dependent monooxygenases. Due to their instability and membrane-bound property, the purification and characterization of these enzymes are quite challenging, particularly for C3H [10]. It was also suggested that the hydroxylation at the 3-position could also occur after *p*-coumaric acid is esterified, which does not generate caffeic acid as the intermediate [8,11]. Recently, genes and enzymes involved in caffeic acid biosynthesis were also reported in the actinomycete *Saccharothrix espanaensis*. A tyrosine ammonia lyase (TAL) encoded by *sam8 *and a microbial C3H encoded by *sam5 *are responsible for the conversion of tyrosine to *p*-coumaric acid and then to caffeic acid, respectively [12].

Currently, caffeic acid is commonly produced by extraction from plant sources, such as coffee beans. Chemical or enzymatic hydrolysis of caffeoylquinic acid derivatives is also employed to produce caffeic acid [13,14]. Like many other secondary metabolites, caffeic acid derivatives are usually accumulated at low levels in plants and hence the isolation of these compounds is to some extent difficult and expensive. Microbial conversion provides an alternative approach to caffeic acid production. Sachan *et al. *reported the co-production of caffeic acid and *p*-hydroxybenzoic acid in *Streptomyces caeruleus *by feeding *p*-coumaric acid [15]. Over decades, advances in metabolic engineering and synthetic biology enable the production of various plant-specific secondary metabolites in recombinant microorganisms [16-19]. Most recently, the conversion of tyrosine to caffeic acid (the titer was not reported) and ferulic acid (7.1 mg/L) in *E. coli *was achieved by the co-expression of the enzymes encoded by the *sam5 *and *sam8 *from *S. espanaensis *and an *O*-methyltransferase from *Arabidopsis thaliana *[20] . However, the above-mentioned studies relied on feeding the direct precursors such as tyrosine and *p*-coumaric acid, which would increase the production cost and can not be preferred for large-scale production. Alternatively, the development of processes that can enable the biosynthesis of these high-value metabolites from simple carbon sources is much more desirable. By utilizing tyrosine-overproducing strains as hosts, the production of several natural compounds such as *L*-dopa, flavonoids, and benzylisoquinoline alkaloids from simple carbon sources has already been achieved [21-23].

**Figure 1:**
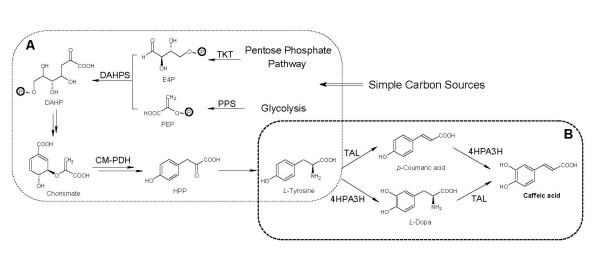
**Proposed caffeic acid biosynthetic pathway**. (A) Native tyrosine biosynthetic pathway in *E. coli*. (B) The artificial dual pathway mediated by 4HPA3H and TAL for caffeic acid biosynthesis from tyrosine. PPS: phosphoenolpyruvate synthase; TKT: transketolase; CM-PDH: chorismate mutase-prephenate dehydrogenase; DAHPS: 3-deoxy-D-arabino- heptulosonate-7-phosphate synthase; 4HPA3H: 4-hydroxyphenylacetate 3-hydroxylase; TAL: tyrosine ammonia lyase; E4P: *D*-erythrose-4-phosphate; PEP: phosphoenolpyruvate; HPP: 4-hydroxyphenylpyruvate.

In this study, we characterized the *E. coli *native 4-hydroxyphenlacetate 3-hydroxylase (4HPA3H) that was capable of hydroxylating *p*-coumaric acid and tyrosine in addition to its native substrate 4-hydroxyphenylacetic acid. Moreover, we found the TAL from *Rhodobacter capsulatus *was able to accept both tyrosine and *L*-dopa as substrates. Based on these findings, we further designed a novel dual pathway leading from tyrosine to caffeic acid mediated by the enzymes 4HPA3H and TAL. As shown in Figure 1B, native tyrosine biosynthesis can be extended by the introduction of the 4HPA3H and TAL, yielding *L*-dopa and *p*-coumaric acid, respectively. Then TAL further converts *L*-dopa to caffeic acid; while 4HPA3H converts *p*-coumaric acid into caffeic acid as well. Furthermore, by grafting this dual pathway into *E. coli*, we successfully achieved *de novo *biosynthesis of caffeic acid. This work not only opens the route to the production of caffeic acid from simple carbon sources, but also paves the way to the microbial synthesis of many other phenylpropanoids derived from caffeic acid.

## Results and discussion

Plants and bacteria are very different in cell structure, physiology and genetics. One of the difficulties in reconstructing plant pathways in microbial systems is the availability of functional enzymes that are compatible with the specific microorganism. For the biosynthesis of caffeic acid in plants, two cytochrome P450-dependent monooxygenases are involved, which are C4H and C3H [10]. Due to the requirement for anchorage on endoplasmic reticulum, functional expression of these plant P450-dependent enzymes were always problematic in bacterial systems [10,24]. Fortunately, TALs identified from various sources can catalyze the direct formation of *p*-coumaric acid from tyrosine bypassing the enzymatic step catalyzed by C4H [25], and thus, the need for a C4H was not obligatory. Nevertheless, the need for C3H still remains. Although an alternative microbial C3H was identified from *S. espanaensis*, its activity seems to be low, which limits its applications [20].

### *p*-Coumaric Acid Hydroxylation by 4HPA3H

**Figure 2:**
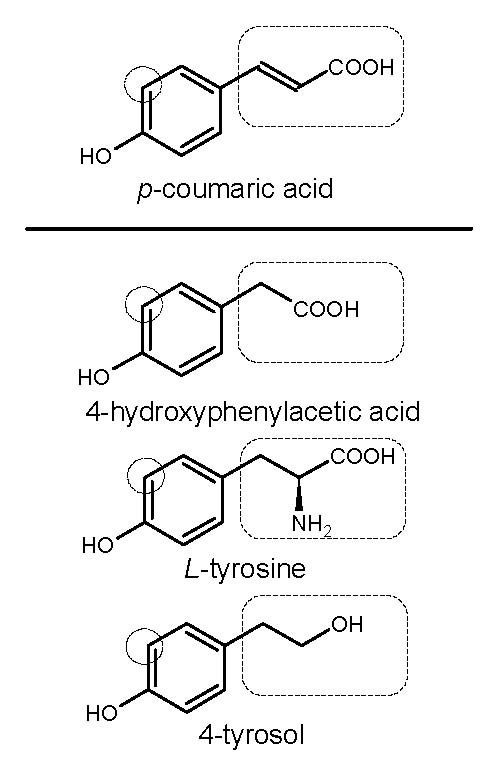
**Molecular structures of *p*-coumaric acid and three known substrates of 4HPA3H**. The circles indicate the hydroxylation positions. The boxes indicate the difference of molecular structures.

One of the most challenging steps in reconstructing plant phenylpropanoid pathway in *E. coli *is the 3-hydroxylation of *p*-coumaric acid, because all C3Hs identified in plants are cytochrome P450-dependent monooxygenases and are hard to be functionally expressed in bacterial systems [10]. Therefore, the exploration of alternative enzymes compatible with *E. coli *is necessary. By examining *E. coli *native enzymes and pathways related to metabolism of aromatic compounds, we reasoned that the 4HPA3H complex encoded by the operon *hpaBC *involved in the 4-hydroxyphenylacetate (4-HPA) degradation may play the role of C3H [26,27]. This enzyme complex can accept a broad range of substrates and has been applied to produce *L*-dopa and hydroxytyrosol from tyrosine and 4-tyrosol, respectively [23,26-28]. Because *p*-coumaric acid is similar to 4-HPA, tyrosine and 4-tyrosol in molecular structure (Figure 2), we reasoned that the catalytic pocket of 4HPA3H should be able to accommodate *p*-coumaric acid as well.

To test this hypothesis, we first cloned *hpaBC *into a high-copy expression vector pZE12-luc. After over-expressing this enzyme complex in wild type *E. coli *BW25113, crude extract was prepared for *in vitro *enzyme assay. Our results indicate that 4HPA3H complex is capable of converting *p*-coumaric acid to caffeic acid in the presence of flavin adenine dinucleotide (FAD) and nicotinamide adenine dinucleotide hydride (NADH) (Table 1). Its specific activity toward *p*-coumaric acid (5.37 × 10^-3 ^U/mg protein) is much higher than its activity toward tyrosine (2.44 × 10^-3 ^U/mg protein).

**Table 1:** *In vitro *activity of 4HPA3H complex

Enzyme	Activity toward Substrate		Ratio (A : B)
	**A (tyrosine)** **(10^-3 ^U/mg protein)**	**B (*p*-coumaric acid)** **(10^-3 ^U/mg protein)**	

4HPA3H	2.44 ± 0.11	5.37 ± 0.31	0.45

One U (unit) is defined as the amount (1 μmole) of product formed per minute.

**Figure 3:**
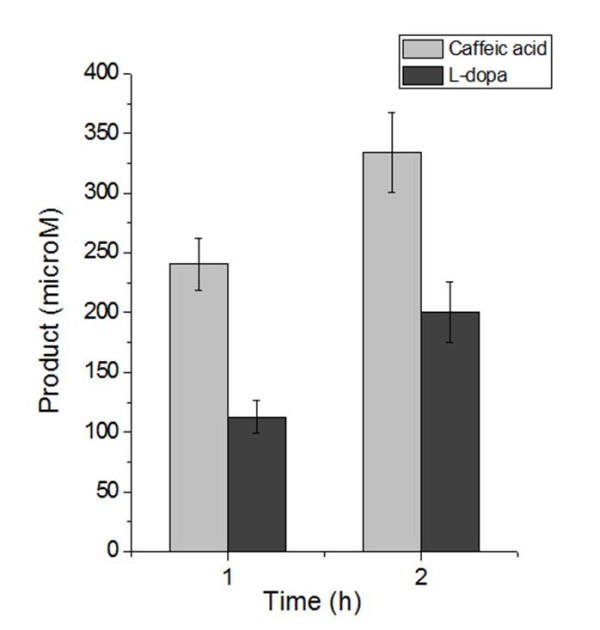
***In vivo *enzyme activity of 4HPA3H complex toward tyrosine and *p*-coumaric acid**. The grey and black bars refer to the amount of caffeic acid and *L*-dopa, respectively.

Furthermore, we carried out whole-cell conversion studies which reflect the *in vivo *enzymatic activity. BW25113 harboring pZE-*Ec*HpaBC was able to completely convert 100 mg/L *p*-coumaric acid to caffeic acid within 3 hours after the induction of isopropyl *β*-*D*-1- thiogalactopyranoside (IPTG), indicating the *in vivo *activity toward *p*-coumaric acid is high. Meanwhile, no caffeic acid was detected in the culture of the control strain (BW25113 harboring pZE12-luc) even after 20 hours. This phenomenon suggested that although *hpaBC *exists in the genome of *E. coli*, it is not natively expressed. Thus, over-expression of *hpaBC *is necessary to obtain adequate 4HPA3H activity. The result of *in vivo *enzyme assay showed that the highest conversion rates (within the first hour) from tyrosine to *L*-dopa and from *p*-coumaric acid to caffeic acid are 112.98 and 240.80 μmol· h^-1^·gDCW^-1^, respectively (Figure 3). For both products, we did not observe obvious intracellular accumulation. Both *in vitro *and *in vivo *assay results indicate that *p*-coumaric acid is preferred by 4HPA3H. To our knowledge, this is the first report of the 4HPA3H activity toward *p*-coumaric acid.

### Comparison of *Rc*TAL and *Rs*TAL

Previous studies reported that TALs from *R. capsulatus *(*Rc*) and *R. sphaeroides *(*Rs*) catalyze the deamination of tyrosine [25]. In addition, *Rs*TAL can also take *L*-dopa as a substrate [29]. But the activity of *Rc*TAL toward *L*-dopa has not been investigated. To evaluate the performance of the two TALs in *E. coli*, we performed *in vitro *enzyme assays using crude extracts. The genes encoding the two TALs were cloned and expressed in *E. coli *using the plasmids pZE-*Rc*TAL and pZE-*Rs*TAL. Interestingly, both TALs slightly prefer *L*-dopa over their native substrate tyrosine. For *Rc*TAL, the specific activities toward tyrosine and *L*-dopa were 0.93 × 10^-3 ^and 1.54 × 10^-3 ^U/mg protein, respectively. For *Rs*TAL, the specific activities are 0.80 × 10^-3 ^and 1.14 × 10^-3 ^U/mg protein, respectively. The results indicated that *Rc*TAL is slightly more active than *Rs*TAL toward both substrates (Table 2). As a control, the crude extract of the wild-type *E. coli *carrying the blank vector did not exhibit any activity.

**Table 2:** Comparison of *in vitro *activity of *Rc*TAL and *Rs*TAL

Enzyme	Activity toward Substrate*		Ratio (A : C)
	**A (tyrosine)** **(10^-3 ^U/mg protein)**	**C (*L*-dopa)** **(10^-3 ^U/mg protein)**	

*Rc*TAL	0.93 ± 0.03	1.54 ± 0.05	0.60
*Rs*TAL	0.80 ± 0.13	1.14 ± 0.03	0.70

* The crude extract of the wild-type *E. coli *did not show any TAL activity

### Production of caffeic Acid in *E. coli*

**Figure 4:**
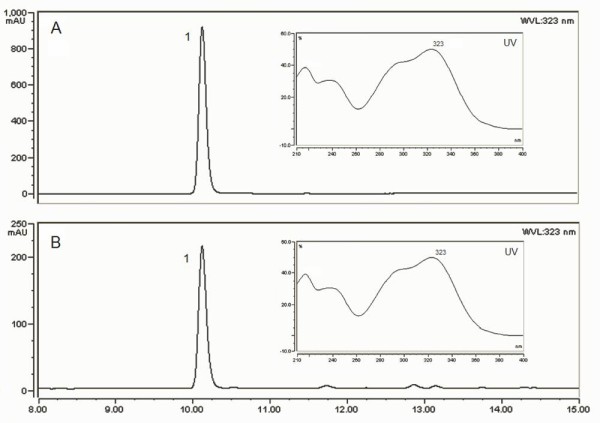
**HPLC analysis of caffeic acid produced by engineered *E. coli *(A) Standard, 50 mg/L caffeic acid**. (B) A sample taken from the fermentation culture of YL-2 after 24 hours. Peak 1 corresponded to caffeic acid. The retention time was 10.1 min. UV absorbance profiles are shown beside the peaks.

Based on the activities of 4HPA3H and *Rc*TAL, we proposed a novel dual pathway for caffeic acid biosynthesis from tyrosine (Figure 1B). Because *E. coli *natively biosynthesizes tyrosine, it is expected that the introduction of *Rc*TAL and 4HPA3H can result in the biosynthesis of caffeic acid by utilizing *E. coli *endogenous tyrosine (Figure 1). To achieve this goal, the genes encoding *Rc*TAL and 4HPA3H were amplified and consecutively cloned into a high-copy-number plasmid pZE12-luc under the control of a strong IPTG-inducible promoter P_L_lacO1, generating the plasmid pZE-TH. A ribosome binding site (RBS) was placed upstream of each gene. Strain YL-2 was developed by introducing pZE-TH into wild type *E. coli *strain BW25113 to test this pathway. The production of caffeic acid was carried out in shake flasks using modified M9 minimal salt medium as described in "Methods and Materials". High performance liquid chromatography (HPLC) analysis of the fermentation samples showed that the retention time (10.1 min) and UV profile of the product were identical to those of the caffeic acid standard, confirming that caffeic acid was produced (Figure 4). The strain YL-2 was able to produce 11.1 ± 1.1 mg/L caffeic acid after 24 hours, without obvious accumulation of intermediates including tyrosine, *p*-coumaric acid and *L*-dopa. 48-hour cultivation did not lead to a great increase in caffeic acid production (12.1 ± 0.3 mg/L) (Table 3). However, *L*-dopa was accumulated at a concentration of 7.4 ± 0.2 mg/L.

**Table 3:** Production of caffeic acid and tyrosine by engineered *E.coli *strains

Strain	24 hours				48 hours			
	
	Product (mg/L)	Intermediates (mg/L)			Product (mg/L)	Intermediates (mg/L)		
				
	caffeic acid	tyrosine	*p*-coumaric acid	*L*-dopa	caffeic acid	tyrosine	*p*-coumaric acid	*L*-dopa
YL-2	11.1 ± 1.1	< 0.2	< 0.2	< 0.2	12.1 ± 0.3	< 0.2	< 0.2	7.4 ± 0.2
YL-2*	20.2 ± 1.8	< 0.2	< 0.2	2.1 ± 0.3	21.5 ± 4.0	< 0.2	< 0.2	14.2 ± 1.7
YL-3	-	188.9 ± 5.7	-	-	-	296.6 ± 1.0	-	-
YL-4	-	218.6 ± 8.2	-	-	-	426.7 ± 4.9	-	-
YL-5	30.5 ± 1.9	17.1 ± 1.8	2.9 ± 0.3	15.4 ± 1.7	50.2 ± 10.1	25.1 ± 2.5	< 0.2	75.3 ± 13.6

-: No production

*: addition of 100 mg/L tyrosine in the cultures

### Construction of tyrosine overproducers

The addition of 100 mg/L tyrosine into the cultures of YL-2 resulted in a two-fold increase in caffeic acid production (Table 3), suggesting that tyrosine is a limiting precursor for caffeic acid biosynthesis. In wild-type *E. coli*, tyrosine biosynthesis is strictly controlled by several regulatory mechanisms. Two feedback inhibition-sensitive enzymes chorismate mutase-prephenate dehydrogenase (CM-PDH, encoded by *tyrA*) and 3-deoxy-*D*-arabino-heptulosonate-7-phosphate synthase (DAHPS, encoded by *aroG*) were identified as the major regulatory components in the tyrosine pathway [30]. The feedback inhibition resistant (fbr) variants *tyrA^fbr ^and aroG^fbr ^*have already been developed and applied in tyrosine production [30,31]. In addition, the availability of erythrose-4-phosphate (E4P) and phosphoenolpyruvate (PEP) is extremely critical to tyrosine biosynthesis (Figure 1A). Over-expression of PEP synthase (PPS, encoded by *ppsA*) and transketolase (TKT, encoded by *tktA*) was able to increase the availability of PEP and E4P, and redirect the carbon flux into the tyrosine pathway [31]. In this work, *tyrA^fbr^*, *ppsA*, *tktA *and *aroG^fbr ^*were consecutively cloned into a medium-copy-number plasmid pCS27 under the control of P_L_lacO1 promoter as well, generating the plasmid pCS-TPTA. By introducing pCS-TPTA into wild type *E. coli *BW25113, we obtained a recombinant strain YL-3. Compared with wild type strain which produced little tyrosine, YL-3 was able to produce 296.6 ± 1.0 mg/L tyrosine in 48 hours, which indicated that over-expression of the four enzymes was effective. Furthermore, the strain YL-1 (Δ*tyrR*) was also employed as the host to alleviate the *tyrR*-mediated regulation [23]. The introduction of pCS-TPTA into YL-1 (yielding strain YL-4) resulted in the accumulation of higher amount of tyrosine (426.7 ± 4.9 mg/L in 48 h, Table 3). It should be noted that YL-4 exhibited only slight improvement in tyrosine production compared to YL-3 in the first 24 hours. However, its advantage was demonstrated in the following 24 hours. These results are consistent with what were reported previously [30,31].

### Improvement of caffeic acid production by tyrosine overproducing strains

Although YL-4 was able to produce higher amount of tyrosine, this *tyrR*-deleted strain seemed to be in conflict with pZE-derived plasmids for unknown reasons and did not express the enzymes 4HPA3H and *Rc*TAL as well as expected. Only a trace amount of caffeic acid (< 0.2 mg/L) and *p*-coumaric acid (< 1 mg/L) but a large amount of tyrosine (> 400 mg/L) were detected in the YL-6 (YL-1 harboring pZE-TH and pCS-TPTA) cultures. Thus, we employed wild type *E. coli *BW25113 as the parent strain. By transforming it with both pZE-TH and pCS-TPTA, we generated the strain YL-5. The titer of caffeic acid in the shake flask fermentation using YL-5 reached 50.2 ± 10.1 mg/L after 48 h fermentation which is a 5-fold increase compared to YL-2. Moreover, we analyzed the intermediates accumulated in the culture. The presence of 25.1 ± 2.5 mg/L tyrosine indicated that tyrosine availability is no longer the limiting factor for caffeic acid production in the strain YL-5. The accumulation of a large amount of *L*-dopa (75.3 ± 13.6 mg/L) and a small amount of coumaric acid (< 0.2 mg/L) suggested that *Rc*TAL became into the rate-limiting step in this artificial pathway, especially after 24 h (Table 3).

## Conclusions

We have successfully established a novel pathway and constructed an *E. coli *strain for the *de novo *production of caffeic acid via metabolic engineering approaches. We first identifed that 4HPA3H can function as a C3H which exhibited decent activity toward *p*-coumaric acid and tyrosine, thus gains great potential for metabolic engineering and biocatalysis applications. In addition, we compared the TALs from *R. capsulatus *(*Rc*) and *R. sphaeroids *(*Rs*) that are able to catalyze the deamination of both tyrosine and L-dopa. RcTAL exhibited higher activities toward both substrates. Then a dual pathway leading from tyrosine to caffeic acid was proposed and introduced into *E. coli*. The artificial pathway extended the native tyrosine pathway of *E. coli *and produced 12.1 mg/L of caffeic acid from simple carbon sources. Further improvement of production was accomplished via alleviating feedback inhibition and redirecting carbon flux into tyrosine biosynthesis. Finally, the titer of caffeic acid reached 50.2 mg/L in shake flasks after 48-hour cultivation.

The established pathway obviated the use of two cytochrome P450-dependent monooxygenases (C4H and C3H) and achieved the *de novo *biosynthesis of caffeic acid, which opened the possibility of microbial synthesis of more complex plant secondary metabolites derived from caffeic acid. However, for the production system to be more economically viable, productivity has to be further improved. We have identified that *Rc*TAL is the rate-limiting enzyme in the pathway once the tyrosine availability issue was solved. To meet the process metrics and avoid the accumulation of the intermediates (tyrosine and *L*-dopa), we will explore more TALs for higher catalytic activity via bioprospecting and protein engineering approaches. In addition to the tyrosine overproducers we generated in this study, the strains employed to produce tyrosine in amino acid industry are also ready to be used as hosts for caffeic acid production. With proper process optimization, industrially relevant production should be expectable.

## Materials and methods

### Chemicals and enzymes

The following commercially available chemicals and enzymes were used in this study: *L*-dopa (ACROS Organics); tyrosine (Sigma-Aldrich), caffeic acid (TCI), *p*-coumaric acid (MP Biochemicals), IPTG (Zymo Research Co.), restriction enzymes (NEB), Hot Start KOD Plus DNA polymerase (EMD Chemicals Inc.), Rapid DNA ligase Kit (Roche). All the enzymes were used according to the instructions provided by the manufacturers.

### Strains, plasmids, media, and growth conditions

*E. coli *XL1-Blue (Stratagene) was used for gene cloning and plasmid propagation. Wild type *E. coli *strain BW25113 (*E. coli *Genetic Resource Center) and its derivatives were employed for either enzyme assays or shake flask experiments. Plasmids pZE12-luc and pCS27 were used for gene over-expression in *E. coli *[32,33]. The characteristics of all the strains and plasmids used in this study are described in Table 4. *E. coli *cells for gene cloning, plasmid propagation, and inoculum preparation were grown in Luria-Bertani (LB) medium at 37°C. The fermentation medium was modified M9 minimal salt medium containing (per liter): glycerol (10 g), glucose (2.5 g), NH_4_Cl (1 g), Na_2_HPO_4 _(6 g), KH_2_PO_4 _(3 g), NaCl (0.5 g), MgSO_4_·7H_2_O (2 mmol), CaCl_2_·2H_2_O (0.1 mmol), vitamin B1 (2.0 mg), H_3_BO_3 _(1.25 mg), NaMoO_4_·2H_2_O (0.15 mg), CoCl_2_·6H_2_O (0.7 mg), CuSO_4_·5H_2_O (0.25 mg), MnCl_2_·4H_2_O (1.6 mg), and ZnSO_4_·7H_2_O (0.3 mg). For the strains carrying plasmids, 100 μg/ml of ampicillin, 50 μg/ml of kanamycin and/or 30 μg/ml of chloramphenicol were added if necessary. For all shake flask experiments, 200 μl overnight LB culture was inoculated into 10 ml fermentation medium and grown at 37°C with shaking. After OD_600 _reached 0.4-0.5, IPTG was added into the cultures to a final concentration of 0.2 mM. Then the cultures were transferred to 30°C in a gyratory shaker at 250 rpm. Samples were collected after 24 and 48 hours, and then analyzed by HPLC.

**Table 4:** Strains and plasmids used in this study

Plasmid or Strain	Relevant characteristics	Source
**Plasmids**		
pZE12-luc	ColE1 ori; Amp^R^; P_L_lacO1; *luc*	Lutz et al., 1997
pCS27	p15A ori; Kan^R^; P_L_lacO1; MCS1	Shen et al., 2008
pZE-*Rc*TAL	From pZE12, P_L_lacO1; *tal(Rc)*	This study
pZE-*Rs*TAL	From pZE12, P_L_lacO1; *tal(Rs)*	This study
pZE-*Ec*HpaBC	From pZE12, P_L_lacO1; *hpaB(Ec)-hpaC(Ec)*	This study
pZE-TH	From pZE12, P_L_lacO1; *tal(Rc)*-*hpaB(Ec)-hpaC(Ec)*	This study
pCS-TPTA	From pCS27, P_L_lacO1; *tyrA^fbr^-ppsA-tktA-aroG*^*fb*r^	This study
**Strains**		
XL1-Blue	*recA1 endA1 gyrA96 thi-1 hsdR17 supE44 relA1 lac *[F' *proAB lacI^q^Z *Δ*M15 Tn10 *(Tet^R^)]	Stratagene
BW25113	F-, Δ(*araD-araB*), ΔlacZ (::*rrnB-3*), λ-, *rph-1*, Δ(*rhaD-rhaB*), *hsdR*	Yale CGSC
JW1316-1	BW25113, Δ*tyrR*::*kan*	Yale CGSC
YL-1	BW25113, Δ*tyrR*::FRT (as JW1316-1, but *kan^R ^*gene deleted)	This study
YL-2	BW25113 harboring pZE-TH	This study
YL-3	BW25113 harboring pCS-TPTA	This study
YL-4	YL-1 harboring pCS-TPTA	This study
YL-5	BW25113 harboring pZE-TH and pCS-TPTA	This study
YL-6	YL-1 harboring pZE-TH and pCS-TPTA	This study

### Molecular biology techniques

General molecular biology techniques and DNA manipulations were carried out according to the standard protocols [34]. Deletion of kanamycin resistant gene from *E. coli *JW1316-1 was conducted using the method described by Kirill A. Datsenko and Barry L. Wanner [35]. Host cells were transformed with the plasmids by electroporation (EPPENDORF Electroporator 2510, 1.8 kV when using 0.1 cm cuvettes).

### Construction of plasmids

To construct pZE-*Rc*TAL and pZE-*Rs*TAL, the genes encoding *Rc*TAL and *Rs*TAL were amplified by high-fidelity polymerase chain reaction (PCR) from the genomic DNAs of *Rhodobacter capsulatus *and *Rhodobacter sphaeroides *using the primers listed in Additional file 1: Table S1 [25]. Amplified fragments and pZE12-luc were digested with *Kpn*I and *Sph*I, and then ligated with Rapid DNA ligase. To construct pZE-*Ec*HpaBC, the gene cluster *hpaBC *was amplified from *E. coli *MG1655 genome directly. The amplified *hpaBC *fragment was inserted into pZE12-luc vector between *Kpn*I and *Sph*I as well. The pZE-TH was constructed by cloning the gene cluster *hpaBC *into the pZE-*Rc*TAL using restriction enzymes *Sph*I and *Xba*I. A ribosome binding site is located upstream of each gene to facilitate protein expression. The genes *tyrA*, *aroG, ppsA*, and *tktA *were all amplified from *E. coli *MG1655 genomic DNA. Point mutations were introduced to *tyrA *(Met-53-Ile and Ala-354-Val) and *aroG *(Asp-146-Asn) by splicing and overlapping extension PCR (SOE-PCR), generating *tyrA^fbr ^*and *aroG^fbr ^*[36,37]. The genes *tyrA^fbr ^*and *ppsA *were first cloned into pCS27 simultaneously via three-piece ligation using restriction enzymes *Kpn*I, *Nde*I, and *Sal*I, generating the plasmid pCS-TP. Similarly, *tktA *and *aroG^fbr ^*were then simultaneously inserted into pCS-TP using restriction enzymes *Xho*I, *Sph*I, and *Hind*III resulting in pCS-TPTA.

### 4HPA3H *In vitro *assay

The *E. coli *strain BW25113 carrying the plasmid pZE-*Ec*HpaBC was pre-inoculated into LB liquid medium containing 100 μg/ml of ampicillin and grown at 37°C overnight with shaking at 250 rpm. In the following day, 1 ml of preinoculum was added to 50 ml of fresh LB medium also containing 100 μg/ml of ampicillin. The culture was left to grow at 37°C till OD_600 _reached 0.6 and then induced with 0.5 mM IPTG. Protein expression was conducted at 30°C for another 3 h. The cells were harvested and resuspended in 2 ml of buffer A (20 mM KH_2_PO_4_, pH = 7.0), and then lysed by French Press. The soluble fraction was collected by ultra-centrifugation and used as crude enzyme extract for the enzyme assay. Total protein concentration was estimated using the BCA kit (Pierce Chemicals). The total protein concentration of the crude extract is around 6172 μg/ml. The enzyme activity was assayed according to the protocol described by Tai. et al. with a few modifications [26]. The 1 ml reaction system contained 2 mM NADH, 2 mM FAD, 2 mM substrate (tyrosine or *p*-coumaric acid) and 100 μl of crude enzyme extract in buffer A. The reaction was incubated at 30°C for 1.5 min and terminated by adding 50 μl HCl (20%) to the 1 ml reaction system. The amount of products (*L*-dopa and caffeic acid, respectively) were measured and quantified by HPLC.

### Whole-cell Bioconversion by 4HPA3H

The *E. coli *strain BW25113 carrying the plasmid pZE-*Ec*HpaBC was pre-inoculated into LB liquid medium containing 100 μg/ml of ampicillin and grown at 37°C overnight with shaking at 250 rpm. Then 0.1 ml of preinoculum was added to 10 ml of fresh LB medium also containing 100 μg/ml of ampicillin. The culture was grown at 37°C till OD_600 _reached 0.6 and then induced with 0.5 mM IPTG for 3 hours. After that, 100 uL of *p*-coumaric acid (10 g/L) was added to reach a final concentration of 100 mg/L. Samples were collected at 3 h and analyzed by HPLC

### 4HPA3H *In vivo *assay

The pre-inoculum of *E. coli *strain BW25113 carrying pZE-*Ec*HpaBC from an overnight culture was added in to 10 ml of LB medium (1:100 V/V) and grown at 37°C. IPTG was added to the cultures to a final concentration of 0.5 mM until OD_600 _reached 0.6. The cultures were left at 30°C for around another 3 hours with shaking for protein expression till OD_600 _reached 3.0 (approximately equivalent to 1 g/L cell). Then the cells were collected, washed, resuspended in 10 ml of NaCl (0.9%) solution. 1 mM substrate (tyrosine or *p*-coumaric acid) was added to the cell resuspensions at 30°C. Samples were collected after 1 and 2 hours, and then analyzed by HPLC.

### Enzyme assay of *Rc*TAL and *Rs*TAL

The crude enzyme extracts of *Rc*TAL and *Rs*TAL were prepared as described before [25]. But the cells were resuspended in buffer B (50 mM, Tris-HCl, pH = 8.5). The 1 ml reaction system contained 2 mM substrate (tyrosine or *L*-dopa) and 100 μl crude extract in buffer B. The reaction was incubated at 30°C for 1.5 min and the amount of products (*p*-coumaric acid and caffeic acid, respectively) was measured by HPLC.

### HPLC analysis of products

Tyrosine, *L*-dopa, *p*-coumaric acid, and caffeic acid generated in enzyme assays and fermentations were quantitatively analyzed by HPLC (Dionex Ultimate 3000) with a reverse-phase ZORBAX SB-C18 column and an Ultimate 3000 Photodiode Array Detector. The compounds were separated by elution with a methanol-water gradient (water containing 0.2% trifluoroacetic acid). The following gradient was used at a flow rate of 1 ml/min: 10 to 50% methanol for 15 min, 50 to 10% methanol for 1 min, and 10% methanol for an additional 4 min. Quantification for the four above-mentioned compounds was based on the peak areas of absorbance at 274, 280, 308 and 323 nm, respectively. The data shown in this study were generated from duplicate or triplicate independent experiments.

## Competing interests

The University of Georgia has filed a United State provisional patent on this technology.

## Authors' contributions

YY and YL conceived the study. YL conducted the experiments under the direction of YY. YL did literature review and drafting of the manuscript. YY made revisions. Both authors read and approved the final manuscript.

## Supplementary Material

Additional file 1 **Table S1 Primers used in this study**. Click here for file
