# Supplementary material for: Biosynthesis of caffeic acid in Escherichia coli using its endogenous hydroxylase complex
Source: Microb Cell Fact. 2012 Apr 4;11:42. doi: 10.1186/1475-2859-11-42 (PMC3379962; doi:10.1186/1475-2859-11-42)
Supplement: Additional file 1 — Table S1 Primers used in this study. [file 1475-2859-11-42-S1.DOC]

**Supplementary Materials**

**Table S1.** Primers used in this study

| **Plasmid** | **Gene** | **Sequence (5’-3’)** |
| --- | --- | --- |
| pZE-*Rc*TAL | *tal(Rc)* | F: gggaaaGGTACCatgctcgatgcaaccatcgg |
|  |  | R: gggaaaGCATGCtcatgccgggggatcggc |
| pZE-*Rs*TAL | *tal(Rs)* | F: gggaaaGGTACCatgctcgccatgagccc |
|  |  | R: gggaaaGCATGCtcagacgggagattgctgcaag |
| pZE-*Ec*HpaBC | *hpaBC(Ec)* | F: gggaaaGGTACCatgaaaccagaagatttccgcgc |
|  |  | R: gggaaaGCATGCttaaatcgcagcttccatttccagc |
| pZE-TH | *hpaBC(Ec)* | F: gggaaaGCATGCaggagatataccatgaaaccagaagatttccgcgccag |
|  |  | R: gggaaaTCTAGAttaaatcgcagcttccatttccagcatc |
|  | *tyrA*/Met-53-Ile | F: gcgcgaggcatctattttggcctcgcgtcgtg |
|  |  | R: cacgacgcgaggccaaaatagatgcctcgcgc |
|  | *tyrA*/Ala-354-Val | F: ctggttcggcgattacgtgcagcgttttcagagtg |
|  |  | R: cactctgaaaacgctgcacgtaatcgccgaaccag |
|  | *aroG*/Asp-146-Asn | F: gcaggtgagtttctcaacatgatcaccccac |
|  |  | R: gtggggtgatcatgttgagaaactcacctgc |
| pCS-TPTA | *tyrA^fbr^* | F: gggaaaGGTACCatggttgctgaattgaccgcattacg |
|  |  | R: gggaaaGTCGACgCATATGttactggcgattgtcattcgcctgac |
| pCS-TPTA | *ppsA* | F: gggaaaCATATGaggagatataccatgtccaacaatggctcgtcac |
|  |  | R: gggaaaGTCGACttatttcttcagttcagccaggcttaac |
| pCS-TPTA | *tktA* | F: gggaaaCTCGAGaggagatataccatgtcctcacgtaaagagcttgcc |
|  |  | R: gggaaaGCATGCttacagcagttcttttgctttcgcaac |
| pCS-TPTA | *aroG^fbr^* | F: gggaaaGCATGCaggagatataccatgaattatcagaacgacgatttacgc |
|  |  | R: gggaaaAAGCTTttacccgcgacgcgcttttac |

Capital letters indicate restriction sites; underlines indicate *RBS*
